# Biosynthesis of lactones from diols mediated by an artificial flavin

**DOI:** 10.1186/s40643-021-00450-x

**Published:** 2021-10-01

**Authors:** Xiaowang Zhang, Zhuotao Tan, Chaojian Li, Siyu Qi, Mengjiao Xu, Ming Li, Wenlong Xiong, Wei Zhuang, Dong Liu, Chenjie Zhu, Hanjie Ying

**Affiliations:** 1 College of Biotechnology and Pharmaceutical Engineering, Nanjing Tech University, Nanjing, China; 2 Technology Center, China Tobacco Jiangsu Industry Co., Ltd., Nanjing, 210019 Jiangsu China; 3 School of Life Sciences, Zhengzhou University, Zhengzhou, China

**Keywords:** Lactones, Diols, Cofactor regeneration, Flavin, Biosynthesis

## Abstract

**Background:**

Lactones are important compounds in the field of medicine, material and chemical industry. One of the promising accesses to these flexible scaffolds is NAD(P)^+^-dependent alcohol dehydrogenases-catalyzed oxidative lactonization of diols, which relies on the construction of an efficient NAD(P)^+^ regeneration system.

**Results:**

In this study, a novel system combining horse liver alcohol dehydrogenase (HLADH) with the synthetic bridged flavin cofactor was established for biosynthesis of lactones. The reaction conditions of this system were optimized and a variety of lactones including chiral lactones were efficiently obtained from various diols. Compared to the previously reported NAD(P)^+^-regeneration systems, this system showed better regeneration efficiency and product yield. A two-phase system was further applied to solve the problem of product inhibition, and 80% yield was obtained at the condition of 300 mM substrate.

**Conclusions:**

This study provides an efficient method to synthesis of lactones from diols under mild conditions. We believe this system will be a promising alternative to promote the synthesis of other valuable compounds.

**Graphic abstract:**

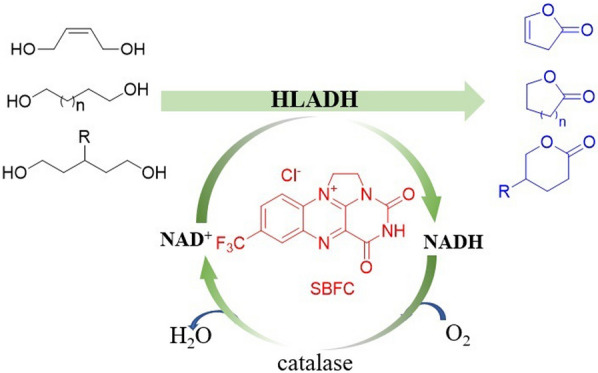

**Supplementary Information:**

The online version contains supplementary material available at 10.1186/s40643-021-00450-x.

## Introduction

**Fig. 1:**
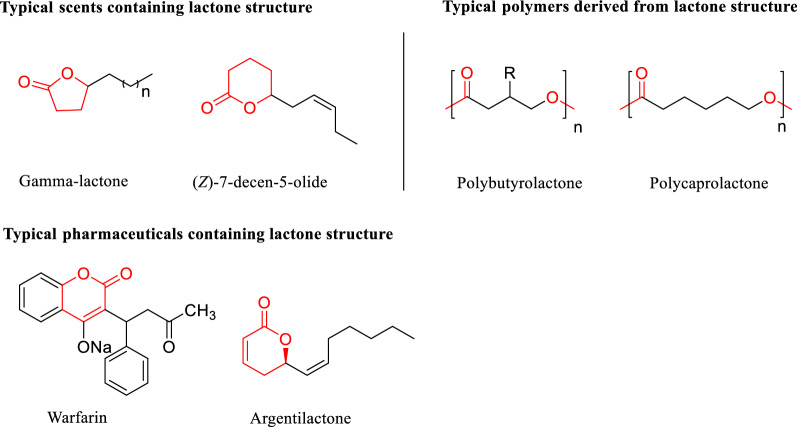
Types of compounds derived from lactone structure

Lactones are key structural motifs in several important chemicals, such as pharmaceuticals, scents and polymers (Fig. 1), due to their excellent physical and biological properties (Delgove et al. 2018; Schulz and Hotling 2015; Kim et al. 2021). Over the past few years, various chemical methods for the production of lactones have been extensively studied, such as esterification of hydroxycarboxylic acid (Kauloorkar and Kumar 2016), lactonization of hydroxy ester under acidic or basic conditions (Marco et al. 2007), lactonization of cyclic ketones by Baeyer–Villiger reaction (Maalouf et al. 2020) and iodolactonization of enoic acid (Nolsøe and Hansen 2014). However, most of them suffer from disadvantages, such as high temperature and pressure, strong acids or bases, rare metal catalysts and stoichiometric amounts of oxidizing agents (Sartori et al. 2021).

**Scheme 1.**
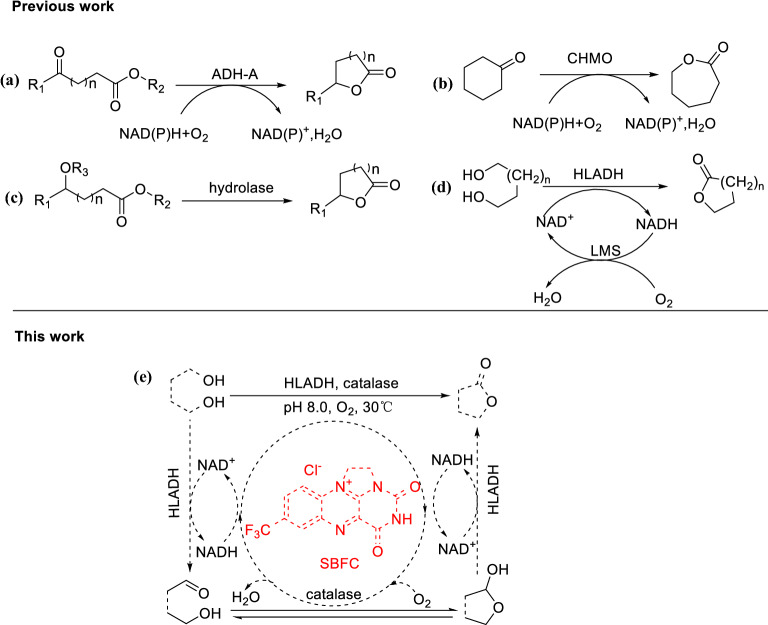
Previous and current strategies for the synthesis of lactones

The development of biosynthetic methods for lactones has captured the attention of the scientific community over the years due to advantages, such as exquisite selectivity, environmental friendliness, and high catalytic efficiency (Puetz et al. 2020; Winkler et al. 2021; Schulz and Hotling 2015; Kim et al. 2021). Several methods for biosynthesis of lactones have been studied (Scheme 1), such as reductive lactonization of ketone ester compounds by ADH (alcohol dehydrogenase) from *Rhodococcus ruber* (ADH-A) (Díaz-Rodríguez et al. 2013) (Scheme 1a), Baeyer–Villiger oxidation of the corresponding cyclic ketones through a monooxygenase (BVMO) (Fink et al. 2013) (Scheme 1b) and lactonization of hydroxy esters precursors by hydrolase (Kamal et al. 2003) (Scheme 1c). Recently, horse liver alcohol dehydrogenase (HLADH)-catalyzed oxidative lactonization of diols was reported (Kara et al. 2013) (Scheme 1d), a laccase-mediator system was used for the NAD^+^ cycle in the reaction system. However, side reaction of hydrolysis of the generated lactone products was observed as a major limiting factor toward application of this system.

Recently, we have reported a NAD(P)^+^ regeneration system using synthetic bridged flavin catalyst (**SBFC**) (Zhu et al. 2016; Tan et al. 2018). In this simple and green system, NAD(P)^+^ could be effectively regenerated in vitro or in vivo using molecular oxygen as terminal electron acceptor. We wonder if this strategy can be applied for the oxidative lactonization reaction. Herein, we established a method for the HLADH-catalyzed transformation of diols into lactones mediated by **SBFC** (Scheme 1e).

## Materials and methods

### Chemicals and strains

The flavin catalysts **SBFC**, 3-phenyl-1,5-pentanediol and 3-(4-methoxyphenyl)-1,5-pentanediol was synthesized according to the previous report (Zhu et al. 2016). Detailed methods are described in Additional file 1: Additional methods. Other reagents were ACS reagent grade and used without further purification unless otherwise noted. Catalase were purchased from Aladdin Reagent. *Escherichia coli* (*E. coli*) DH5ɑ and *E. coli* BL21 (DE3) cells were purchased from Stratagene (La Jolla, CA, USA).

### Gene synthesis and construction of recombinant strains

The genes of alcohol dehydrogenase (ADH) (gene ID: 100034242) from horse liver and myoglobin (Mb) (gene ID: 100054434) from horse heart were both synthesized by GENEWIZ (Genewiz Biotech Co. Ltd. China) with codons optimized for expression in *E. coli* BL21*.* The gene of HLADH was further cloned into the NdeI/HindIII sites of pET28a to give pET28a-HLADH plasmid and the gene of Mb was further cloned into the NdeI/XhoI sites of pET21a to give pET21a-Mb plasmid.

### Enzyme preparation, purification and assay

First, the recombinant *E. coli* BL21 were cultured in LB medium (10 mL) supplemented with 50 μg mL^−1^ kanamycin (HLADH)/100 μg mL^−1^ ampicillin at 37 °C for 12 h. Then 10 mL cell solutions were inoculated to 400 mL LB medium supplemented with 50 μg mL^−1^ kanamycin/100 μg mL^−1^ ampicillin until the OD_600_ value increased to 0.6–0.8. Finally, the cells were induced with 0.1 mM isopropyl-β-d-1-thiogalactopyranoside (IPTG) for 20 h at 25 °C and then harvested by centrifugation at 4 °C for 5 min at 10,000 rpm. Cells of pET28a-HLADH and pET21a-Mb were suspended in phosphate buffered saline (PBS) buffer (50 mM, pH 8.0) and disrupted by an Ultrasonic Cell Disruptor (JY92-II, Scientz Biotech. Co. Ltd.). Cell supernatant was separated from the cell lysate by centrifugation at 4 °C for 15 min at 10,000 rpm. The purified protein was then obtained by gradient elution of cell supernatant with different concentration of imidazole–PBS buffer at a flow rate of 3 mL min^−1^ using a Protein Purifier (GE AKTA Pure) equipped with a His trap Ni–NTA FF column. The protein concentration was determined by the Bradford method using bovine serum albumin as standard.

### HLADH-catalyzed oxidation of diols coupled with SBFC

A mixture of diol (20 mM), NAD^+^ (0.1 mM), **SBFC** (0.05 mM) and catalase (2 μM) were prepared in 50 mM Tris–HCl buffer (pH 8.0). The reactions were initiated by the addition of HLADH solution (0.3 g L^−1^) in a total of 1 mL of aqueous medium. Reaction mixtures (1 mL) were shaken at 300 rpm in 2 mL centrifuge tube vessels at 30 °C. Aliquots (200 µL) were taken at intervals and mixed with 1000 µL of ethyl acetate. The mixture was vortexed for 30 s, followed by centrifugation (12,000 rpm, 2 min). The organic phase was obtained and dried over anhydrous MgSO_4_, which was further identified by GC–MS (gas chromatography–mass spectrometry).

### Different concentrations of substrate catalyzed by HLADH–SBFC system

A mixture of 1,4-butanediol (1,4-BD) (10 mM–250 mM), NAD^+^ (0.1 mM), **SBFC** (0.05 mM) and catalase (2 μM) were prepared in 50 mM Tris–HCl buffer (pH 8.0). The reactions were initiated by the addition of HLADH solution (0.3 g L^−1^) in a total of 1 mL of aqueous medium. Reaction mixtures (1 mL) were shaken at 300 rpm in 2 mL centrifuge tube vessels at 30 °C. The samples (200 µL) were taken at intervals and 1000 µL of ethyl acetate were added. The mixture was vortexed for 30 s, followed by centrifugation (12,000 rpm, 2 min). The organic phase was obtained and dried over anhydrous MgSO_4_, which was further identified by GC–MS (gas chromatography–mass spectrometry).

### HLADH-catalyzed oxidation of 1,4-BD coupled with myoglobin (Mb)

A mixture of 1,4-BD (250 mM), NAD^+^ (0.1 mM), Mb (0.2 g L^−1^) and H_2_O_2_ (500 mM) were prepared in 50 mM Tris–HCl buffer (pH 8.0). The reactions were initiated by the addition of HLADH solution (0.3 g L^−1^) in a total of 1 mL of aqueous medium. Reaction mixtures (1 mL) were shaken at 300 rpm in 2 mL centrifuge tube vessels at 30 °C. The samples (200 µL) were taken at intervals and 1000 µL of ethyl acetate were added. The mixture was vortexed for 30 s, followed by centrifugation (12,000 rpm, 2 min). The organic phase was obtained and dried over anhydrous MgSO_4_, which was further identified by gas chromatography–mass spectrometry (GC–MS).

### HLADH-catalyzed oxidation of 1,4-BD in a two-liquid phase system (2LPS)

A mixture of 1,4-BD (100–300 mM), NAD^+^ (0.1 mM), **SBFC** (0.05 mM) and catalase (2 μM) was prepared in 50 mM Tris–HCl buffer (pH 8.0). The reactions were initiated by the addition of HLADH solution (0.3 g L^−1^) in a total of 1 mL of aqueous medium. After addition of the enzymes, 1 mL of organic solvent was immediately added to the aqueous reaction medium. The reaction mixtures (2 mL) were shaken at 300 rpm in 10 mL falcon tubes at 30 °C. Aliquots (25 µL) were taken from the clearly separated organic phase at intervals and mixed with 225 µL of ethyl acetate. The mixture was vortexed for 20 s and dried over anhydrous MgSO_4_, which was further identified by GC–MS.

### Analytical method

Characterization of all substrates and products was analyzed by GC–MS instrument (Agilent 7890B GC/5977A MS detector) which was equipped with an HP-5 MS capillary column (30 m × 0.25 mm × 0.25 μm). The injection volume was 1.0 μL with an autosampler and helium was used as a carrier gas with column flow rate of 1.5 mL min^−1^. The electron ionization (EI) mass spectra in the range of 35–700 (*m/z*) were recorded in the full-scan mode. The detected compounds were identified based on NIST database. The products except 4-phenyltetrahydro-2*H*-pyran-2-one (**8b**) and 4-(4-methoxyphenyl) tetrahydro-2*H*-pyran-2-one (**9b**) are quantified by gas chromatography. GC was equipped with an HP-INNOWAX capillary column (60 m × 0.25 mm × 0.5 μm) or a modified β-cyclodextrin capillary column CP-Chirasil DEX CB (25 m × 0.25 mm × 0.25 μm) for chiral separation. The products **8b** and **9b** were analyzed using High Performance Liquid Chromatography (HPLC) (Agilent 1200) which was equipped with a Chiralcel AS-H capillary column (250 mm × 4.6 mm × 5 μm). Detailed methods are described in Additional file 1: Additional methods.

## Results and discussion

### HLADH-SBFC-catalyzed oxidative lactonization of 1,4-BD

**Fig. 2:**
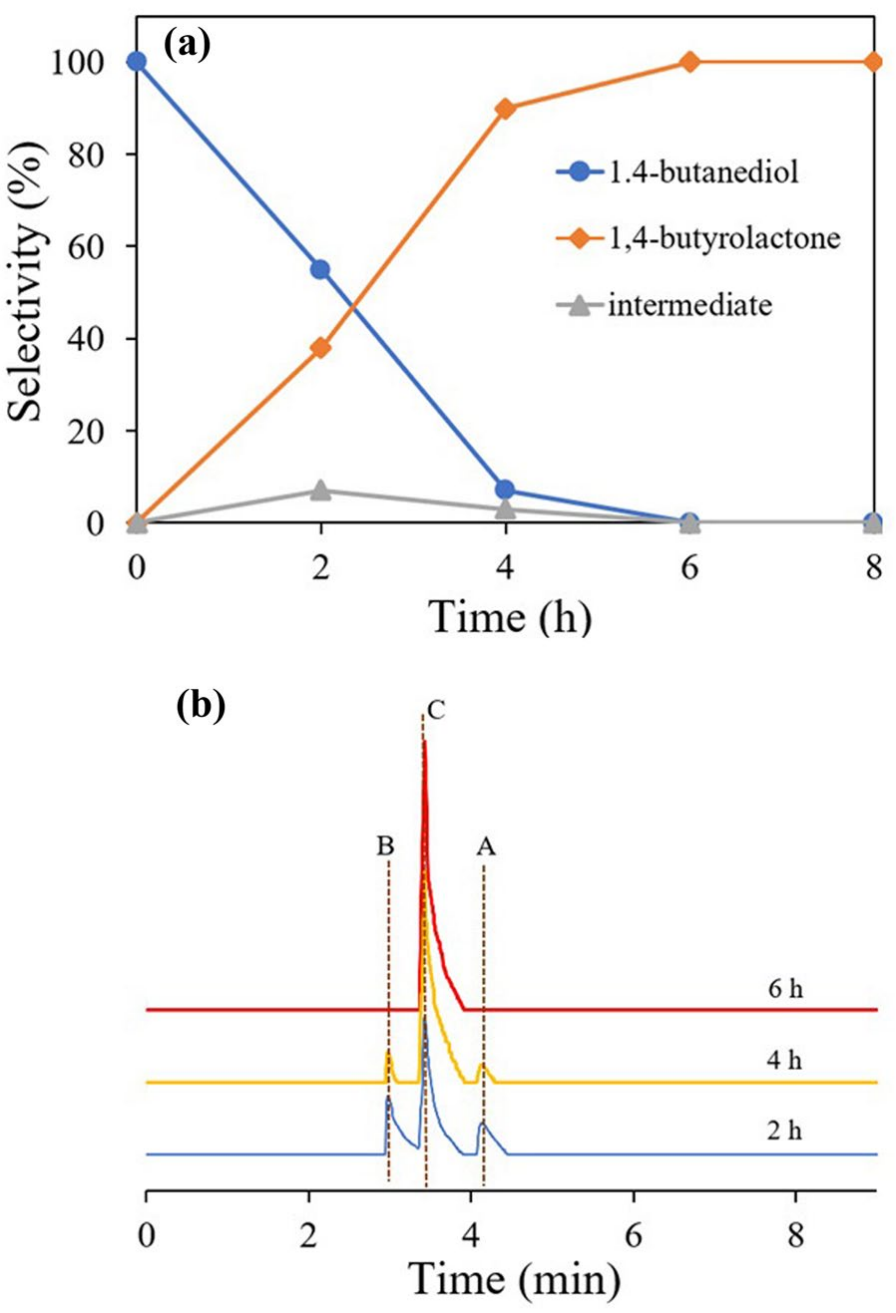
HLADH–**SBFC**-catalyzed oxidative lactonization of 1,4-BD. **a** Time-selectivity plots of HLADH-catalyzed oxidation of 1,4-BD. 1,4-BD (20 mM), NAD^+^ (0.5 mM), **SBFC** (0.1 mM), HLADH (0.3 g L^−1^), and catalase (20 U/mL) in 1 mL of aqueous Tris–HCl buffer (50mM, pH 8.0) at 30 °C. **b** The results of reactions after 2 h, 4 h and 6 h detected by GC–MS. Peak A: 1,4-BD; Peak B: lactol; Peak C: butyrolactone

In the first set of experiments, 1,4-BD was used as the model substrate. As shown in Fig. 2a, > 99% yield of butyrolactone was obtained in 6 h, revealing that this HLADH-**SBFC** system-catalyzed oxidative lactonization was successfully constructed. Time-selectivity plots of 1,4-BD conversion showed that lactol was generated during the reaction and disappeared at the end of the reaction (Fig. 2b). It indicated that there were three steps in the oxidative lactonization of 1,4-BD to butyrolactone. HLADH first oxidized 1,4-BD to an unstable hydroxy aldehyde intermediate, which then spontaneously cyclized to the corresponding intermediate lactol, the subsequent further oxidation of lactol gave access to the stable final product lactone (Scheme 1). The control experiments proved that in the absence of HLADH, NAD^+^ or **SBFC**, no significant conversion of 1,4-BD was detected. In addition, catalase was added to the reaction to eliminate the produced H_2_O_2_ which was generated as a by-product in the NAD^+^ regeneration system.

Optimization of the whole system was further investigated. First, considering the cost of cofactor NAD^+^, we explored if decreasing the amount of NAD^+^ could affect the efficiency of the reaction. It was found that there was no effect on the conversion of 1,4-BD when decreased the amount of NAD^+^ from 0.5 to 0.1 mM (Table 1, entries 1 and 2). Further decreasing the amount to 0.05 mM, it took twice as long as the original catalytic time to finish this reaction (Table 1, entry 3). With regard to the dosage of **SBFC**, it was possible to decrease the amount of **SBFC** to as low as 0.05 mM without a loss in the catalytic efficiency of the reaction (Table 1, entry 4). Further reducing the amount of **SBFC** to 0.01 mM led to a significant decrease of conversion (Table 1, entry 5). The influence of pH and temperature on the reaction were further explored, the results showed HLADH–**SBFC** system exhibited high stability and activity over broad pH (7.0–10.0) and temperature (20 °C to 50 °C) (Additional file 1: Figures S1 and S2). Finally, pH 8.0 and 30 °C were selected as the optical conditions.

**Table 1:** Optimization of HLADH–SBFC system
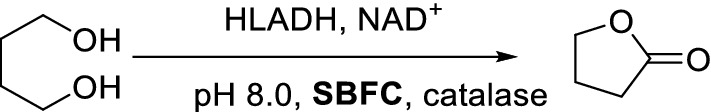

Entry	1,4-BD (mM)	HLADH (mg/mL)	NAD^+^ (mM)	**SBFC** (mM)	Time (h)	Conversion^b^ (%)	Yield^c^ (%)
1^a^	20	0.3	0.5	0.1	6	100	> 99
2	20	0.3	0.1	0.1	6	100	> 99
3	20	0.3	0.05	0.1	12	100	> 99
4	20	0.3	0.1	0.05	6	100	> 99
5	20	0.3	0.1	0.01	12	60	60

^a^Reaction conditions: 1,4-BD (20 mM), NAD^+^ (0.5 mM), **SBFC** (0.1 mM), HLADH (0.3 g L^−1^), and catalase (20 U/mL) in 1 mL of aqueous Tris–HCl buffer (50mM, pH 8.0) at 30 °C

^b^Conversion was determined by GC analysis

^c^Yield was determined by GC analysis

### Substrate scope expanding

With the aim to develop and define the scope and limitation of the present method, the present catalytic system was then extended for the oxidation of a wide range of diols. Excellent chemoselectivity was observed under the present system, the formation of dialdehydes or over-oxidation to carboxylic acid was not found during the reaction. Although a small amount of lactol intermediates were detected during the reaction, but all disappeared at the end of the reactions except for **5a** (vide infra). For saturated straight chain diols, the catalytic efficiency of the reaction decreases with the extension of the carbon chain (Table 2, entries 1–3). 1,4-BD (**1a**) and 1,5-pentanediol (**2a**) underwent smooth oxidation to the corresponding lactone in excellent yield (Table 2, entries 1 and 2). However, the transformation of 1,6-hexanediol (**3a**) into ε-caprolactone was much more sluggish, reaching only 50% yield after prolonged reaction time (Table 2, entry 3), which probably caused by the fact that the lactol formation is rate-determining step, rather than the poor activity of HLADH toward the 7-membered lactol, due to the fact that lactol accumulation was not observed during the reaction. In addition, unsaturated straight chain diols showed less reactive than saturated ones (Table 2, entry 4 vs. entry 1). It is worth noting that the substrate configuration has a great influence on the activity of the reaction. *Cis*-2-butene-1,4-diol (**4a**) was efficiently converted into the corresponding lactone (**4b**) in excellent yield (95%) in 12 h, whereas only trace amounts of product were obtained using *trans*-2-butene-1,4-diol (**5a**) as the substrate (Table 2, entries 4 vs. 5). This result could be explained by the steric hindrance effect during the transformation of hydroxyl aldehyde into lactol, due to hydroxyl aldehyde accumulation was observed during the reaction (Additional file 1: Figure S7). For cycloalkane diol, although its reactivity is lower than that of chain diols, satisfactory yield could also be obtained after prolonged reaction time (Table 2, entry 6), which showed much higher efficiency compared to the previous report using Ru complex as the regeneration catalyst (72 h vs. 116 h) (Gerhard et al. 1997), due to the fact that TOF (turnover frequency) (21.9 min^−1^) of **SBFC**-catalyzed NADH oxidation was higher than that of Ru complex system (TOF 3.4 min^−1^) (Zhu et al. 2016), thus indicating superior catalytic activity with the synthetic flavin analogue.

Chiral lactones are important building blocks for the chemical synthesis (Miyaoka et al. 2011; Lehr and Fürstner 2012), the oxidative desymmetrization of prochiral diols **7a**–**9a** were then examined to assess the feasibility of the present catalytic system for the construction of chiral lactones. Although high yields (> 95%) were achieved in all three reactions, the reaction time and optical purity of the products varied greatly. The transformation of **7a** was completed within 6 h, which was much faster than that of **8a** and **9a** (36 h and 72 h) (Table 2, entries 7–9), which suggested that the substrate with larger substituent group binds less efficiently in the active site of enzyme pocket. In terms of product chirality, this catalytic system proceeded with stereoselectivity towards the (*S*)-enantiomer, which is in accordance with the rule of preferred stereoselectivity of HLADH (Díaz-Rodríguez et al. 2014; Kara et al. 2013). For the reaction of **7a** and **8a**, as high as > 95% *ee* was observed in the present catalytic system (Additional file 1: Figures S12 and S13). In particular, a significant improvement in the enantioselectivity of **8b** was achieved compared to the previously reported HLADH-lactate dehydrogenase (LDH) system (95% *ee *vs. 74% *ee*) (Díaz-Rodríguez et al. 2014). However, the introduction of methoxy group in the aromatic ring at the *para* position led to a significant decline in the *ee* (Table 2, entry 8 vs. 9), which could owe to the additional interactions between methoxy group and residues in the HLADH active site. Based on the above results, we can hypothesize that the enantioselectivity of this process is affected not only by the size of the substituent but also by its interaction with enzyme. Further investigations clarifying these issues are currently underway. Finally, diethylene glycol (**10a**) (Martin et al. 2020) and diethanolamine (**11a**) were tested for the reaction; however, no conversion was detected due to the poor recognition of HLADH (Table 2, entries 10 and 11), despite their structural analogy with **2a** (Table 2).

**Table 2:**
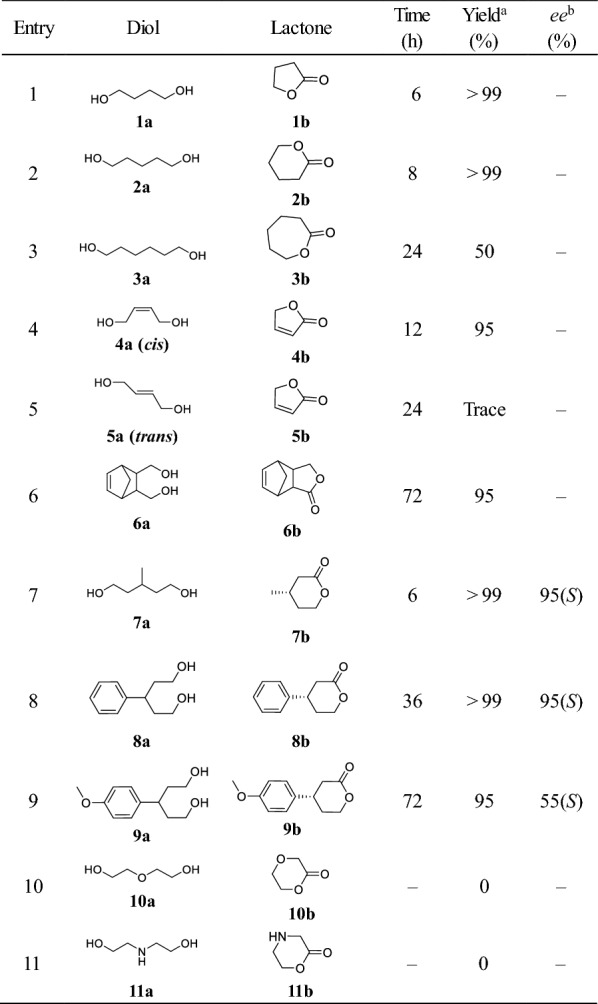
HLADH–SBFC-catalyzed oxidative lactonization reactions

Reaction conditions: diols (20 mM), NAD^+^ (0.1 mM), **SBFC** (0.05 mM), HLADH (0.3 g L^−1^), and catalase (20 U/mL) in 1 mL of aqueous Tris–HCl buffer (50mM, pH 8.0) at 30 °C

^a^Yield were determined by GC and HPLC

^b^The *ee* values of lactones were determined by chiral HPLC and GC

### Scale-up experiment

**Fig. 3:**
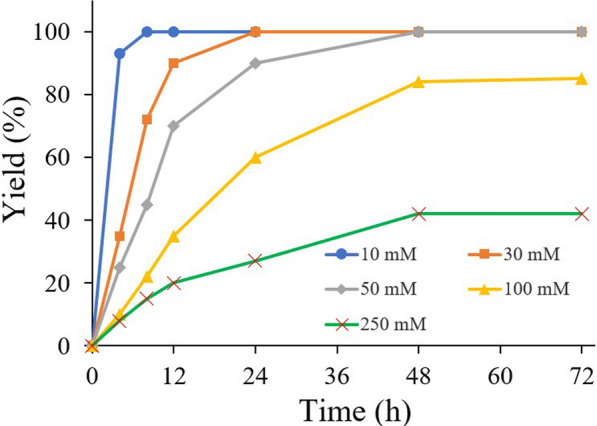
HLADH–**SBFC**-catalyzed oxidation lactonization of 1,4-BD at different substrate concentrations. Reaction conditions: NAD^+^ (0.1 mM), **SBFC** (0.05 mM), HLADH (0.3 g L^−1^), and catalase (20 U/mL) in 1 mL of aqueous Tris–HCl buffer (50mM, pH 8.0) at 30 °C. Yields are average values of duplicates

To assess the possibility of the present catalytic system to more preparative interesting values, the lactonization of 1,4-BD at different concentrations (10–250 mM) were performed under the optimal conditions. As shown in Fig. 3, reasonable yield (80%) could be obtained when the substrate concentration was increased to 100 mM after prolonged reaction time. Further increasing the concentration to 250 mM led to a dramatical decrease in yield (43%). Actually, this phenomenon was also observed in other catalytic systems. We then compared the present **SBFC** system with myoglobin (Mb) system (Jia et al. 2019) and laccase-mediator system (LMS) (Kara et al. 2013) for the oxidative lactonization of 1,4-BD under the condition of 250 mM substrate concentration. As shown in Table 3, only *ca.* 10% yield was obtained using Mb regeneration system after prolonged reaction time (entry 2), this is not only due to the low regeneration efficiency of Mb system, but also due to the inhibition of excessive hydrogen peroxide (Liao et al. 2021). In addition, low yield of 38% was also observed for the LMS regeneration system even under efficient O_2_-intake conditions (entry 3). According to the previous study (Kara et al. 2013), a significant acidification of the reaction mixture was observed for LMS regeneration system, indicating that hydrolysis of γ-butyrolactone occurred during the reaction. This side reaction of hydrolysis was further increased with the increase of laccase dosage, suggesting that the existence of laccase might promote the hydrolysis side reaction. It is worth noting that, compared with LMS regeneration system, hydrolysis of γ-butyrolactone was not found in the present **SBFC** regeneration system, no hydroxy acid formation was detected during the reaction, and the pH of the reaction mixture remained roughly the same even after 72 h. The TOF values of the oxidative lactonization reactions in Table 3 also demonstrated the better catalytic efficiency of HLADH–**SBFC** system.

**Table 3:** HLADH-catalyzed oxidative lactonization reaction mediated by different regeneration systems

Entry	Regeneration system	Yield (%)	Time (h)	TOF^d^ (h^−1^)
1^a^	**SBFC**, O_2_, catalase	43	48	2.23
2^b^	Mb, H_2_O_2_	10	72	0.34
3^c^	Laccase, ABTS, O_2_	38	72	1.32

^a^For **SBFC** regeneration method, **SBFC** (0.05 mM) and catalase (20 U/mL) were supplemented

^b^For Mb regeneration method, Mb (0.2 g L^−1^) and H_2_O_2_ (500 mM) were supplemented

^c^The data come from literature (Kara et al. 2013)

^d^TOF value of catalytic system over the total reaction time. Standard reaction conditions: 1,4-BD (250 mM), NAD^+^ (0.1 mM), **SBFC** (0.05 mM), HLADH (0.3 g L^−1^), and catalase (20 U/mL) in 1 mL of aqueous Tris–HCl buffer (50mM, pH 8.0) at 30 °C

**Fig. 4:**
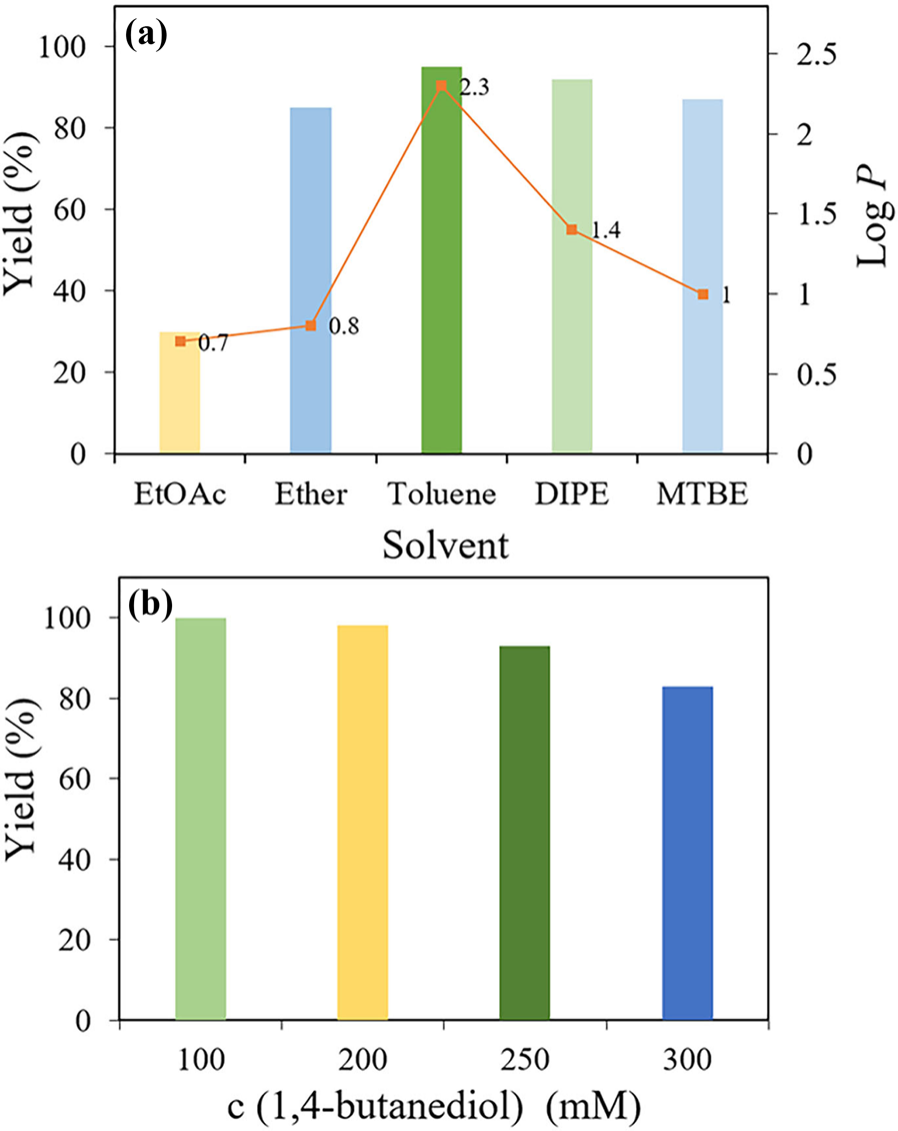
HLADH-catalyzed oxidation lactonization of 1,4-BD in a two-liquid phase system (2LPS). Standard reaction conditions: NAD^+^ (0.1 mM), **SBFC** (0.05 mM), HLADH (0.3 g L^−1^), and catalase (20 U/mL) in 1 mL of aqueous Tris–HCl buffer (50mM, pH 8.0) at 30 °C. **a** 1,4-BD (100 mM), and in different organic phase (5 mL), V_organic_/V_aqueous_ = 5. Detection after 24 h. *EtOAc* ethyl acetate, *DIPE* diisopropyl ether, *MTBE* tert-butyl methyl ether. **b** At different substrate concentrations; organic phase: toluene (1 mL), V_organic_/V_aqueous_ = 1. Detection after 48 h. Yields are average values of duplicates

Excluding the factor of hydrolysis side reaction, we attribute the low yield at high substrate concentration to the product inhibition (Kara et al. 2013). A further attempt using a two-liquid phase system for the in situ extraction of the generated lactone into an organic phase was tried to circumvent this limitation. Five organic solvents which have been reported to have negligible influence on HLADH activity were tested for this reaction (Villela Filho et al. 2003). As shown in Fig. 4a, ethyl acetate exhibited the best extraction efficiency (Additional file 1: Table S1); however, only 30% of yield was observed after the reaction, which probably due to its low log *P* value (0.7) (Villela Filho et al. 2003). Among the selected solvents, toluene gave the highest log *P* value (2.3) and obtained the highest yield (92%). Furthermore, the experiments with different ratios of toluene to water were conducted and the ratio 1:1 was chosen for further investigation due to the favorable yield of lactones and lower amount of toluene in this case (Additional file 1: Figure S15). Thus, a two-liquid phase system of toluene-H_2_O was used for the subsequent experiments. As shown in Fig. 4b, this approach was successfully improved the substrate loading issue. A satisfactory yield of 80% was achieved even at a substrate concentration of 300 mM (Fig. 4b), which is the highest level reported in the literature to date. Particularly worth mentioning is **SBFC** has excellent water solubility due to its organic salt property, which makes it difficult to be extracted into the organic solvent phase (Additional file 1: Table S1), thus avoiding hindering the reaction. This feature makes it extremely suitable for the present two-phase system.

## Conclusion

In conclusion, an efficient biocatalytic oxidation system for the synthesis of lactones from diols mediated by **SBFC** was developed, broad substrate scope, mild reaction conditions, good reaction selectivity, and satisfactory production yield were observed for the present system. Valuable chiral lactones can be obtained with high efficiency. Furthermore, this system showed good organic solvent compatibility and a two-liquid phase system was successfully used to alleviate the lactone inhibition issue, leading to high product yield (80%) at substrate concentration of 300 mM using toluene as the extracting solvent. We believe this method offers an ideal strategy for more related biocatalytic oxidation systems.

### Supplementary Information

**Additional file 1. **Additional method, figures and tables. **Figure S1**. HLADH-catalyzed oxidation of 1,4-butanediol under different pH. **Figure S2.** Effect of temperature on reaction. **Figure S3.** Oxidative lactonization of 1,4-butanediol. **Figure S4.** Oxidative lactonization of 1,5-pentanediol. **Figure S5.** Oxidative lactonization of 1,6-hexanediol. **Figure S6.** Oxidative lactonization of *cis*-2-butene-1,4-diol. **Figure S7.** Oxidative lactonization of *trans*-2-butene-1,4-diol. **Figure S8.** Oxidative lactonization of 5-norbornene-2,3-dimethanol. **Figure S9.** Oxidative lactonization of 3-methyl-1,5-pentanediol. **Figure S10.** Oxidative lactonization of 3-Phenyl-1,5-pentanediol. **Figure S11.** Oxidative lactonization of 3-(4-Methoxyphenyl)-1,5-pentanediol. **Figure S12.** Chiral value of 7b detection by GC. **Figure S13.** Chiral value of 8b detection by HPLC. **Figure S14.** Chiral value of 9b detection by HPLC. **Figure S15.** Effect of ratio (V_aqueous_/V _organic_) on reaction. **Figure S16.** ^1^H-NMR, ^13^C-NMR of SBFC. **Figure S17.** ^1^H-NMR, ^13^C-NMR of 8a. **Figure S18.** ^1^H-NMR, ^13^C-NMR of 9a. **Table S1. **Extraction efficient of organic solvents for butyrolactone and SBFC.

## Data Availability

They are included within the article and its Additional files.

## Abbreviations

HLADH: Horse liver alcohol dehydrogenase
IPTG: Isopropyl β-d-1-thiogalactopyranoside
HPLC: High-performance liquid chromatography
SDS-PAGE: Sodium dodecyl sulfate polyacrylamide gel electrophoresis
SBFC: Synthetic bridged flavin cofactor
Mb: Myoglobin
2LPS: Two-liquid phase system
GC–MS: Gas chromatography–mass spectrometry
GC: Gas chromatography
1,4-BD: 1,4-Butanediol
LDH: Lactate dehydrogenase
LMS: Laccase mediated system
TOF: Turnover frequency
PBS: Phosphate buffered saline
ADH: Alcohol dehydrogenase
HPLC: High Performance Liquid Chromatography
