# Supplementary material for: Biosynthesis of lactones from diols mediated by an artificial flavin
Source: Bioresour Bioprocess. 2021 Oct 1;8(1):94. doi: 10.1186/s40643-021-00450-x (PMC10991483; doi:10.1186/s40643-021-00450-x)
Supplement: Supplementary file 1 — Additional file 1. Additional method, figures and tables. Figure S1. HLADH-catalyzed oxidation of 1,4-butanediol under different pH. Figure S2. Effect of temperature on reaction. Figure S3. Oxidative lactonization of 1,4-butanediol. Figure S4. Oxidative lactonization of 1,5-pentanediol. Figure S5. Oxidative lactonization of 1,6-hexanediol. Figure S6. Oxidative lactonization of cis-2-butene-1,4-diol. Figure S7. Oxidative lactonization of trans-2-butene-1,4-diol. Figure S8. Oxidative lactonization of 5-norbornene-2,3-dimethanol. Figure S9. Oxidative lactonization of 3-methyl-1,5-pentanediol. Figure S10. Oxidative lactonization of 3-Phenyl-1,5-pentanediol. Figure S11. Oxidative lactonization of 3-(4-Methoxyphenyl)-1,5-pentanediol. Figure S12. Chiral value of 7b detection by GC. Figure S13. Chiral value of 8b detection by HPLC. Figure S14. Chiral value of 9b detection by HPLC. Figure S15. Effect of ratio (Vaqueous/V organic) on reaction. Figure S16. 1H-NMR, 13C-NMR of SBFC. Figure S17. 1H-NMR, 13C-NMR of 8a. Figure S18. 1H-NMR, 13C-NMR of 9a. Table S1. Extraction efficient of organic solvents for butyrolactone and SBFC. [file 40643_2021_450_MOESM1_ESM.doc]

**Additional file 1**

**Biosynthesis of lactones from diols mediated by an artificial flavin**

Xiaowang Zhang^1^, Zhuotao Tan^1^, Chaojian Li^2^, Siyu Qi^3^, Mengjiao Xu^1^, Ming Li^1^, Wenlong Xiong^3^, Wei Zhuang^1^, Dong Liu^1^*, Chenjie Zhu^1^*, Hanjie Ying^1^

^1^College of Biotechnology and Pharmaceutical Engineering, Nanjing Tech University, Nanjing, China
^2^Technology Center, China Tobacco Jiangsu Industry Co., Ltd., Nanjing, Jiangsu 210019

^3^School of Life Sciences, Zhengzhou University, Zhengzhou, China

Tel: +86-25-86990001; Fax: +86-25-86990001; E-mail: [liudong@njtech.edu.cn](mailto:liudong@njtech.edu.cn), [zhucj@njtech.edu.cn](mailto:zhucj@njtech.edu.cn).

## Additional methods

## Preparation of the artificial flavin.

**Scheme S1**. Preparation of **SBFC**

I) 2-aminoethanol, K_2_CO_3_, EtOH, reflux, 8 h; II) HCOONH_4_, Pd/C, MeOH, 0 ℃, 1h; III) alloxan monohydrate or N-methyl alloxan monohydrate, B(OH)_3_, AcOH, 50 ℃, overnight; IV) SOCl_2_, 50 ℃, 20 h.

**Preparation of the 3-​phenyl-​1,​5-​pentanediol.**

**Scheme S2.** Preparation of 3-​phenyl-​1,​5-​pentanediol

**Preparation of the 3-​(4-​methoxyphenyl)​-​1,​5-​pentanediol.**

**Scheme S3.** Preparation of 3-​(4-​methoxyphenyl)​-​1,​5-​pentanediol

**Analytical method**

method 1:

Conditions: GC (Agilent 7890A) was equipped with a modified β-cyclodextrin capillary column CP-Chirasil DEX CB (25 m×0.25 mm×0.25 μm) for chiral separation. The injection volume was 0.1 μL with an autosampler and nitrogen was used as a carrier gas, pressure = 7.487 psi, total flow = 63.5 mL/min, column flow = 0.54 mL/min, linear velocity = 18.6 cm/s. The temperature program was carried out as follows: initial temperature 90 ℃ for 3 min, then to 110 ℃ at 20 ℃ min^-1^, and maintained at 110 ℃ for 40 min.

method 2:

Conditions: GC (Agilent 7890A) was equipped with a HP-INNOWAX capillary column (60 m×0.25 mm×0.5 μm) for product quantification. The injection volume was 1 μL with an autosampler and nitrogen was used as a carrier gas, pressure = 14.487 psi, total flow =42.3 mL/min, column flow = 0.54 mL/min, linear velocity = 18.6 cm/s. The temperature program was carried out as follows: initial temperature 80 ℃ for 3 min, then to 260 ℃ at 10 ℃ min^-1^, and maintained at 260 ℃ for 5 min.

Method 3:

GC-MS instrument (Agilent 7890B GC/5977A MS detector) which was equipped with a HP-5 MS capillary column (30 m×0.25 mm×0.25 μm). The injection volume was 1.0 μL with an autosampler and helium was used as a carrier gas with column flow rate of 1.5 mL min^-1^. The electron ionization (EI) mass spectra in the range of 35-700 (*m/z*) were recorded in the full-scan mode. The detected compounds were identified based on NIST database. Conditions: The inlet temperature is 250 ℃; The temperature program was carried out as follows: the initial temperature 50 ℃ for 3 min, and increase to 200 ℃ at 10 ℃/min, and maintained at 200 ℃ for 5 min.

Method 4:

Conditions: Mobile phase: n-hexane/isopropanol=7:3, Flow rate=0.5 mL/min, the temperature is 35 ℃, UV detection wavelength is 210 nm.

**Additional Figures**

**HLADH-catalyzed oxidation of 1,4-butanediol under different pH**

**
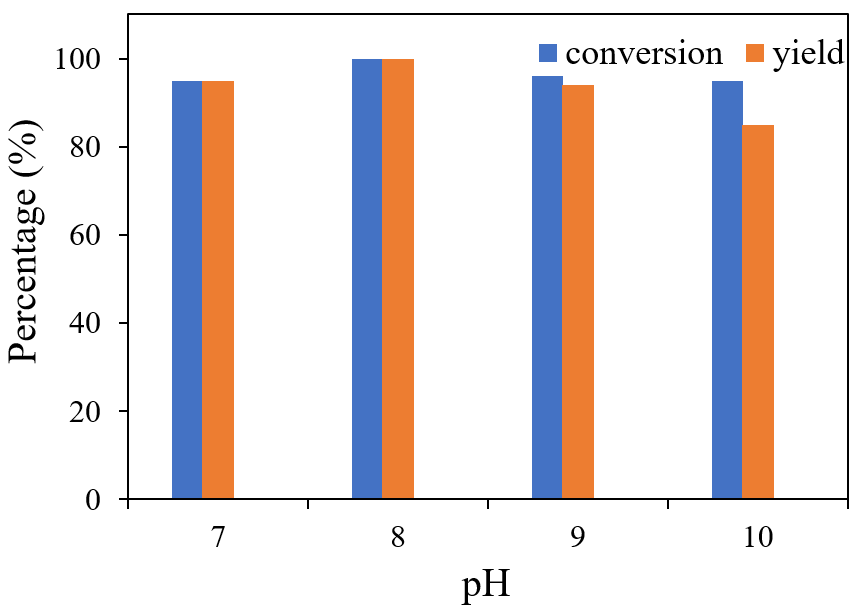
**

**Figure S1** Effect of pH on reaction

**Figure S1** Reaction conditions: 1,4-BD (20 mM), NAD^+^ (0.1 mM), **SBFC** (0.05 mM), HLADH (0.3 g∙L^-1^), and catalase (20 U/mL) in 1 mL (50 mM) of buffer with different pH at 30 °C.

**HLADH-catalyzed oxidation of 1,4-butanediol under different temperature**


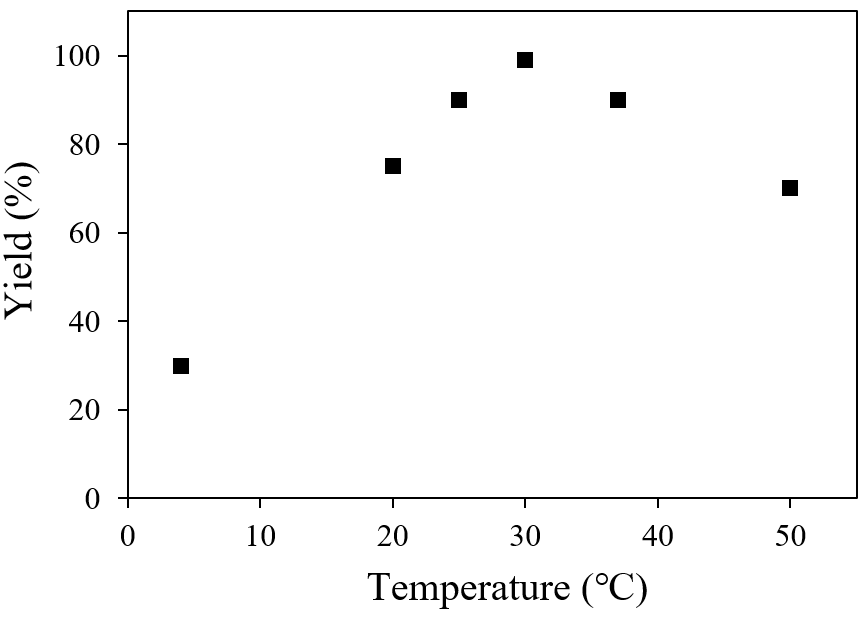


**Figure S2** Effect of temperature on reaction

**Figure S2** Reaction conditions: 1,4-BD (20 mM), NAD^+^ (0.1 mM), **SBFC** (0.05 mM), HLADH (0.3 g∙L^-1^), and catalase (20 U/mL) in 1 mL (50 mM) of aqueous Tris-HCl buffer (pH 8.0). T =4, 25, 30, 37, 50 °C.

**Characterization and analysis of HLADH-catalyzed oxidation of diols with SBFC**

**Oxidative lactonization of 1,4-butanediol (1a)**

**
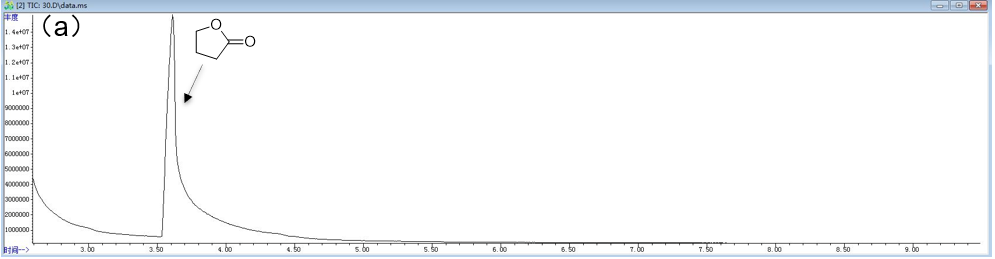
**


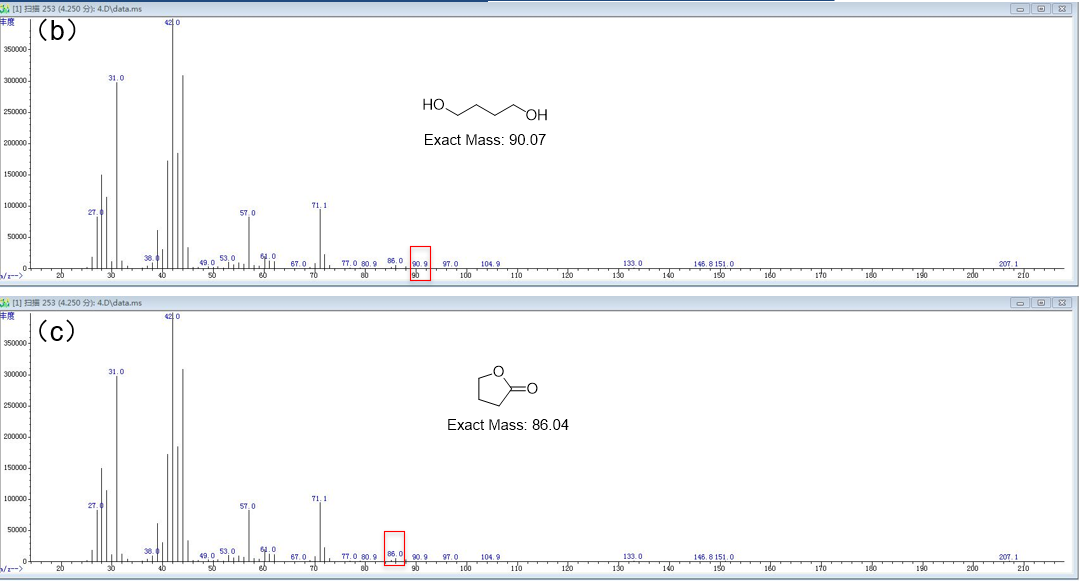


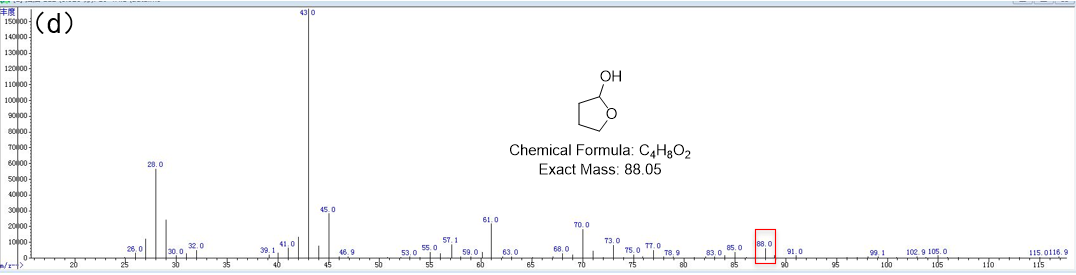


**Figure S3.** a) GC-MS spectrum of the reaction mixture identified after 6 h reaction time; b, c, d) Inset graphics depict the structures of detected compounds based on NIST database; Detection conditions was described in method 3.

**Oxidative lactonization of 1,5-pentanediol (2a)**


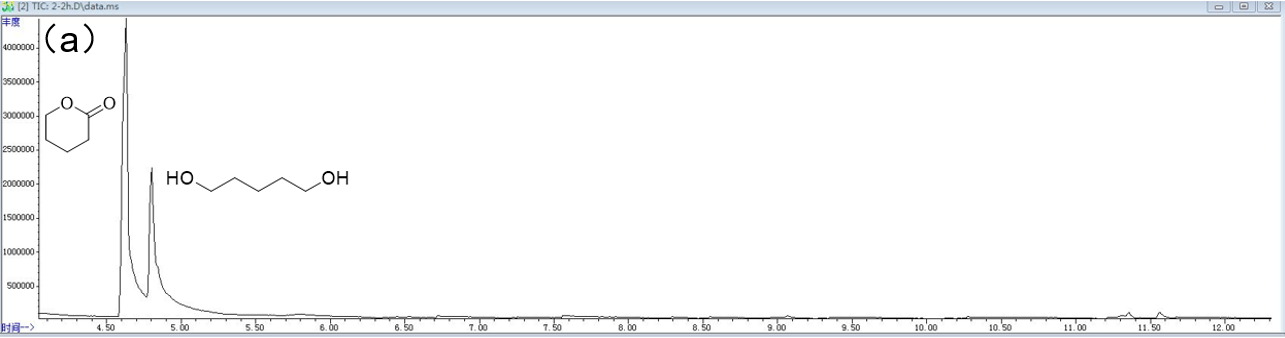


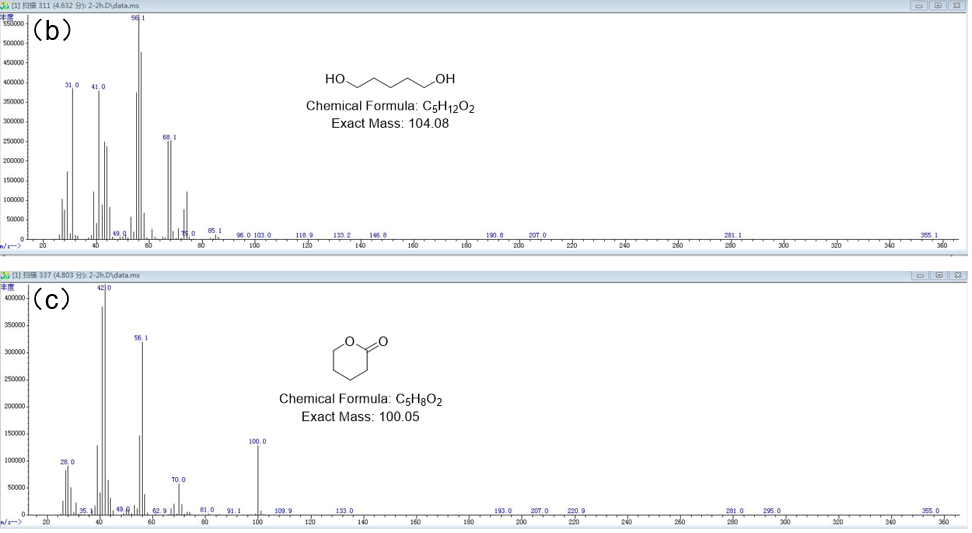


**Figure S4.** a) GC-MS spectrum of the reaction mixture identified after 4 h reaction time; b, c) Inset graphics depict the structures of detected compounds based on NIST database; Detection conditions was described in method 3.

**Oxidative lactonization of 1,6-hexanediol (3a)**


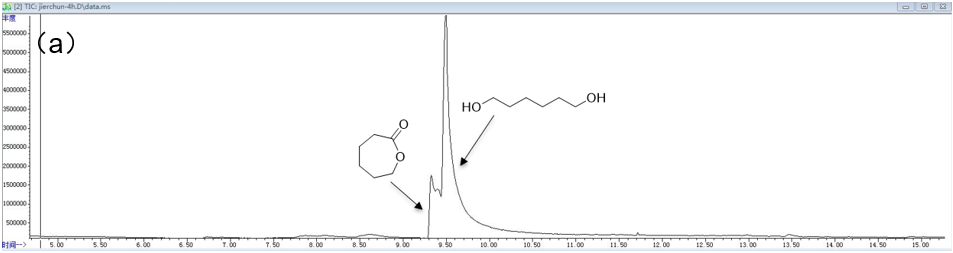


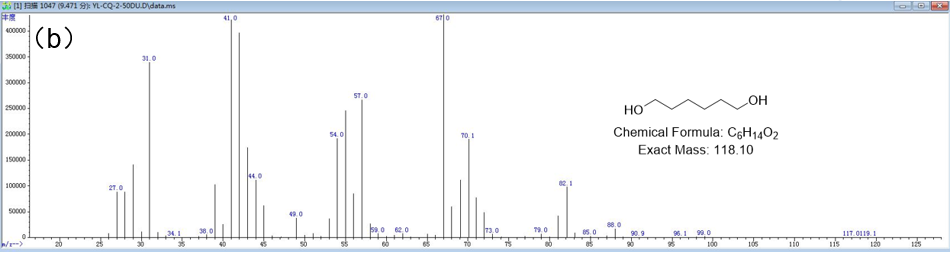


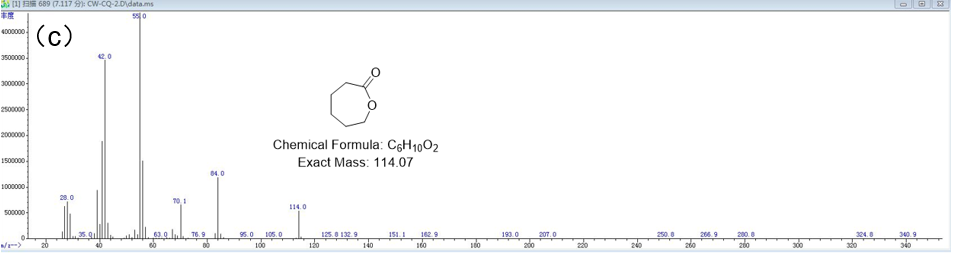


**Figure S5.** a) GC-MS spectrum of the reaction mixture identified after 12 h reaction time; b, c) Inset graphics depict the structures of detected compounds based on NIST database; Detection conditions was described in method 3.

**Oxidative lactonization of cis-2-butene-1,4-diol (4a)**


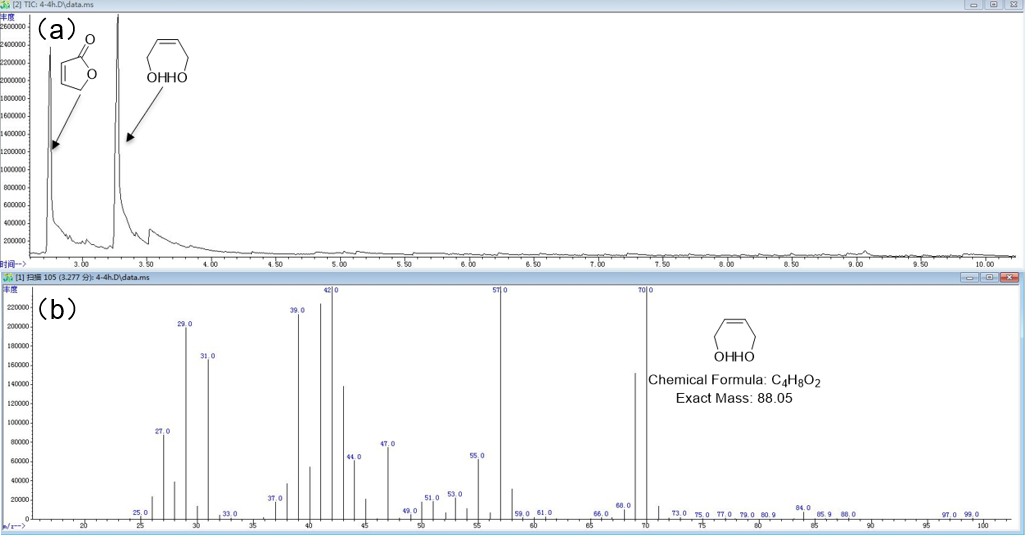


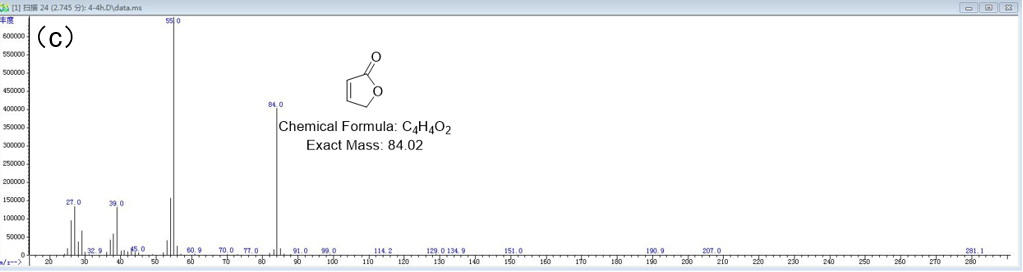


**Figure S6.** a) GC-MS spectrum of the reaction mixture identified after 4 h reaction time; b, c) Inset graphics depict the structures of detected compounds based on NIST database; Detection conditions was described in method 3.

**Oxidative lactonization of trans-2-butene-1,4-diol (5a)**


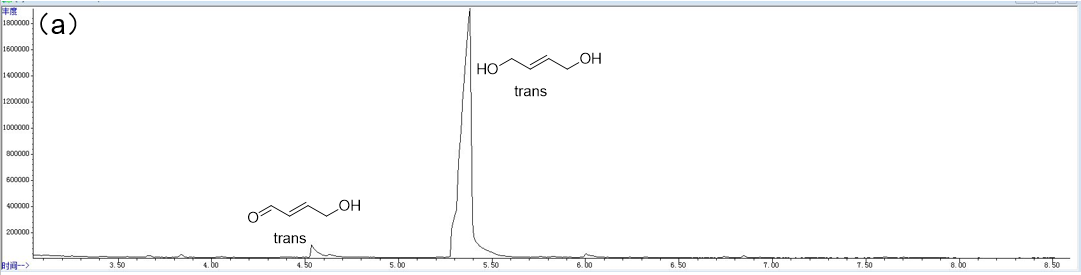


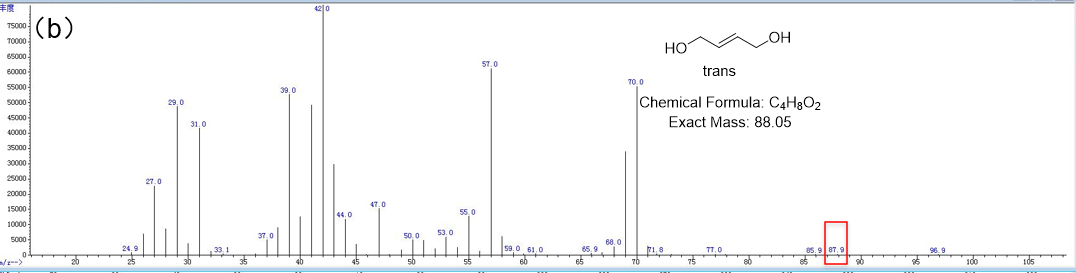


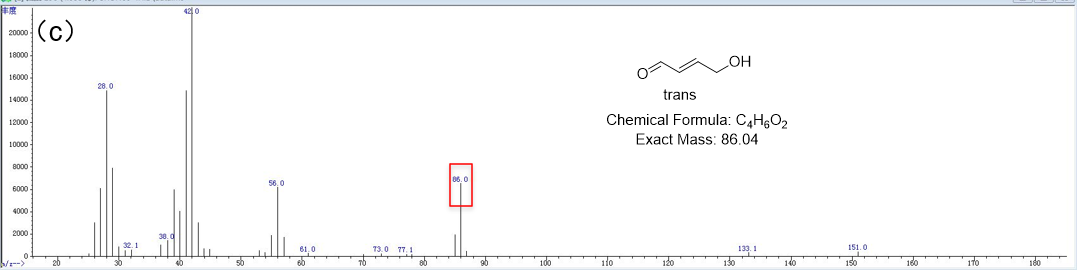


**Figure S7.** a) GC-MS spectrum of the reaction mixture identified after 24 h reaction time; b, c) Inset graphics depict the structures of detected compounds based on NIST database; Detection conditions was described in method 3.

**Oxidative lactonization of 5-norbornene-2,3-dimethanol (6a)**

**
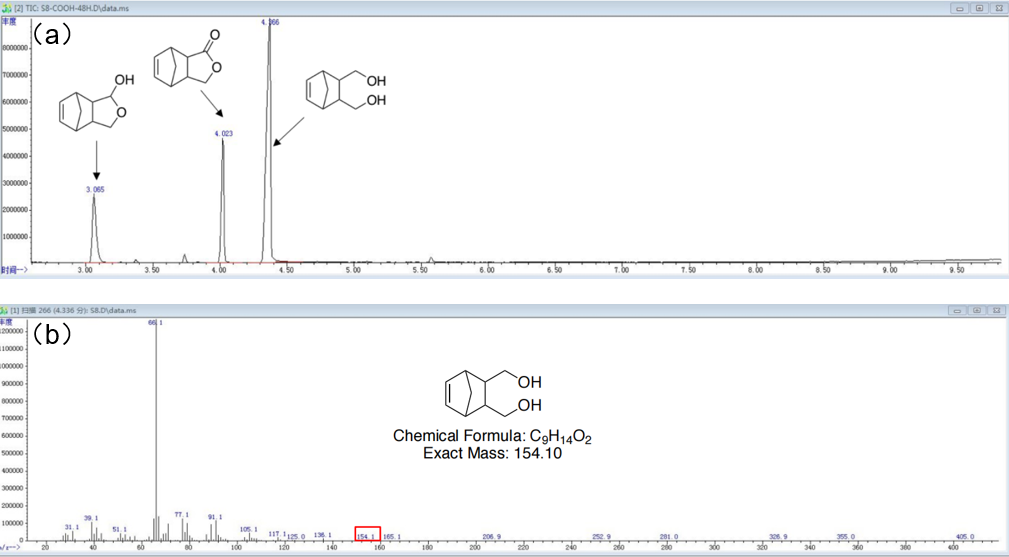
**

**
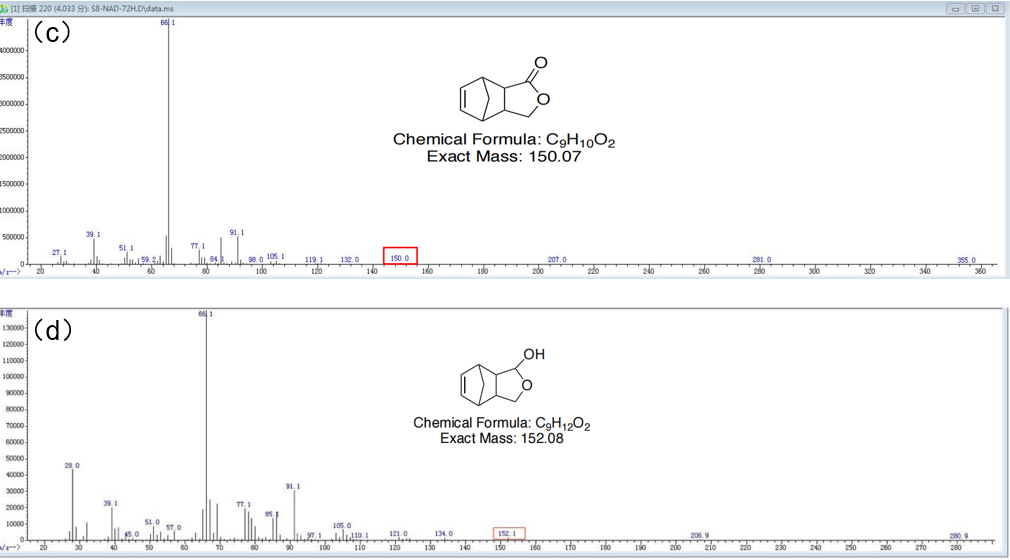
**

**Figure S8.** a) GC-MS spectrum of the reaction mixture identified after 48 h reaction time; b, c, d) Inset graphics depict the structures of detected compounds based on NIST database; Detection conditions was described in method 3.

**Oxidative lactonization of 3-methyl-1,5-pentanediol (7a)**


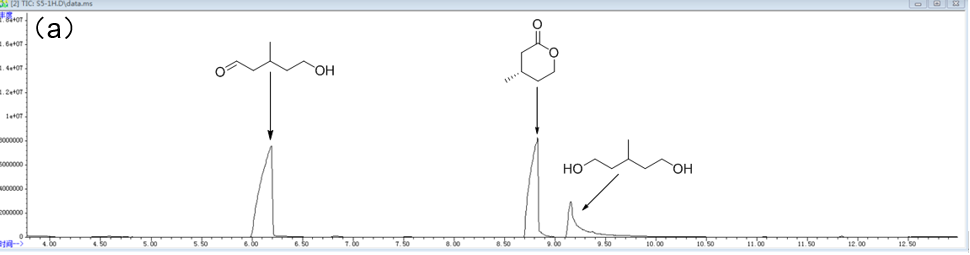


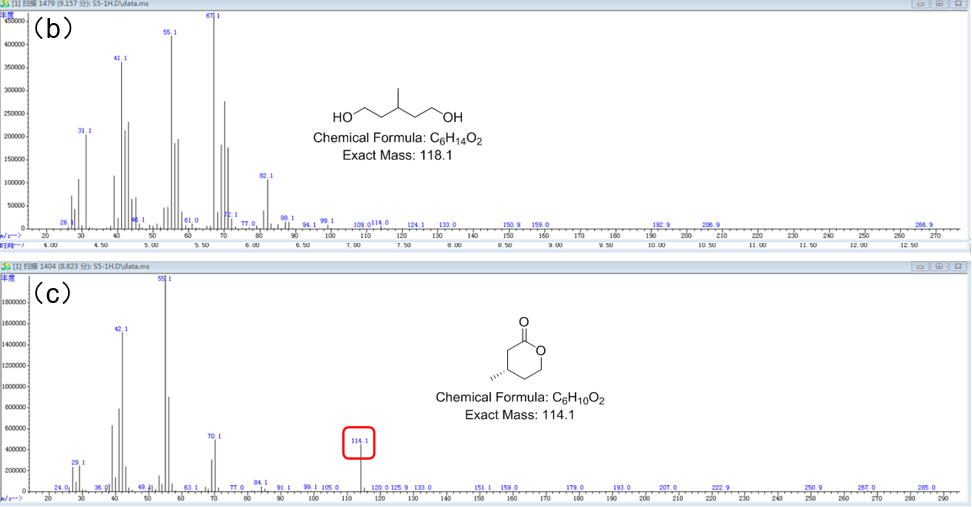


**
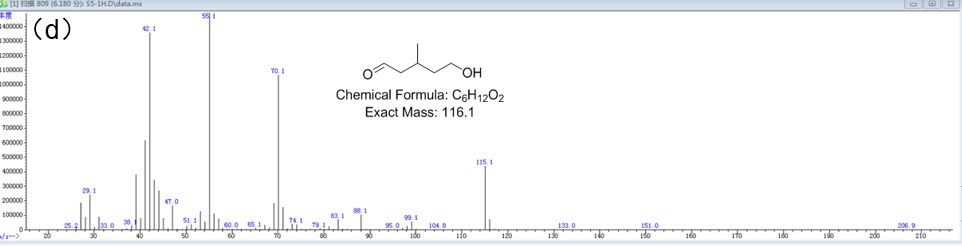
**

**Figure S9.** a) GC-MS spectrum of the reaction mixture identified after 4 h reaction time; b, c, d) Inset graphics depict the structures of detected compounds based on NIST database; Detection conditions was described in method 3.

**Oxidative lactonization of 3-​Phenyl-​1,​5-​pentanediol (8a)**


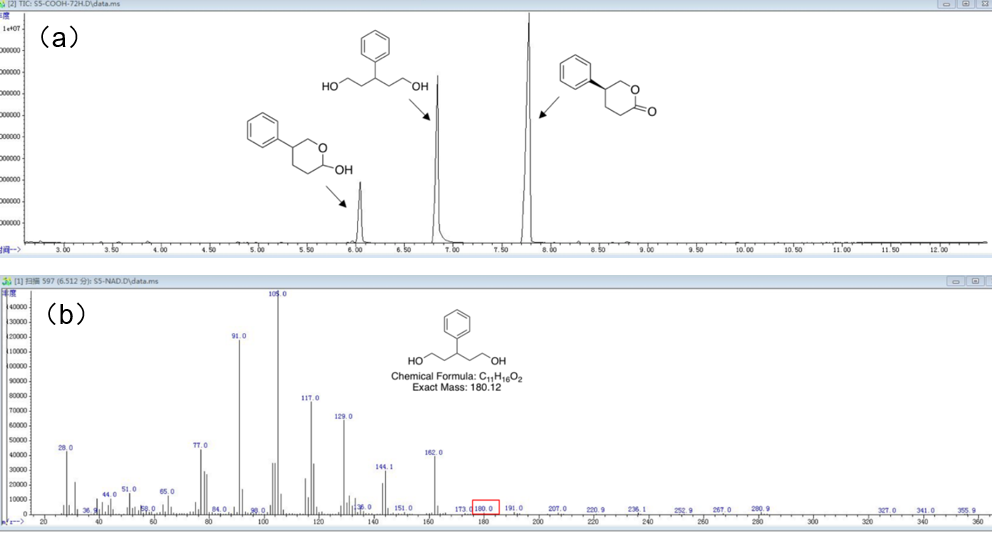


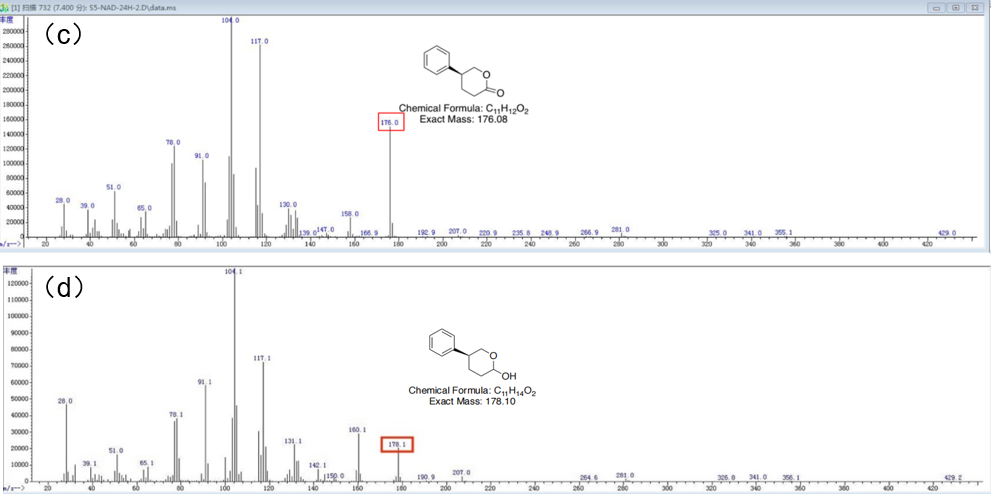


**Figure S10.** a) GC-MS spectrum of the reaction mixture identified after 24 h reaction time; b, c, d) Inset graphics depict the structures of detected compounds based on NIST database; Detection conditions was described in method 3.

**Oxidative lactonization of 3-​(4-​Methoxyphenyl)​-​1,​5-​pentanediol (9a)**

**
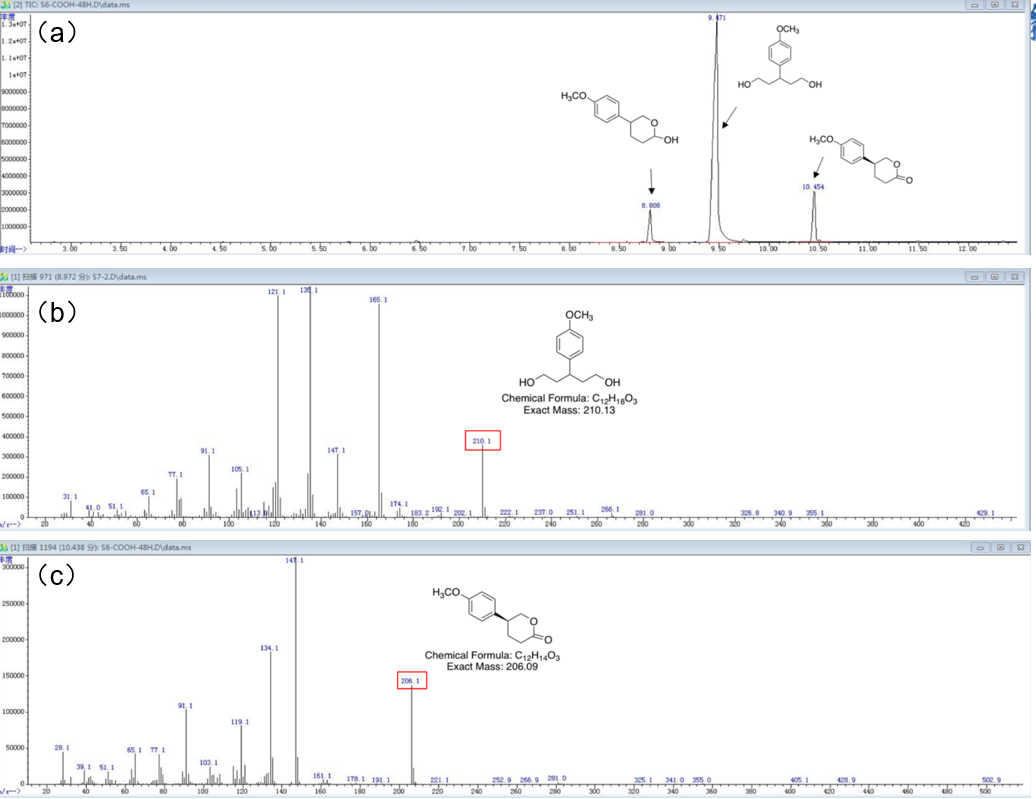
**

**
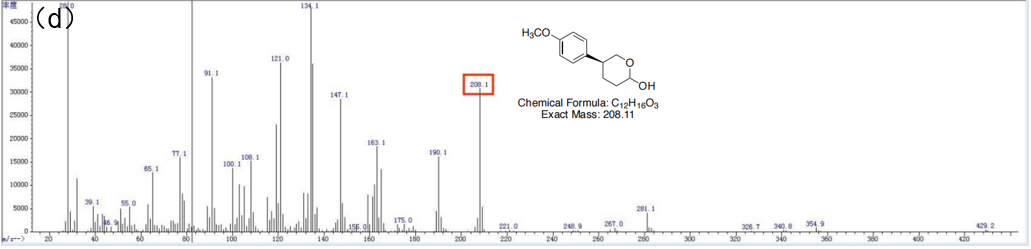
**

**Figure S11.** a) GC-MS spectrum of the reaction mixture identified after 24 h reaction time; b, c, d) Inset graphics depict the structures of detected compounds based on NIST database; Detection conditions was described in method 3.

**Chiral value of 7b detection by GC**

**
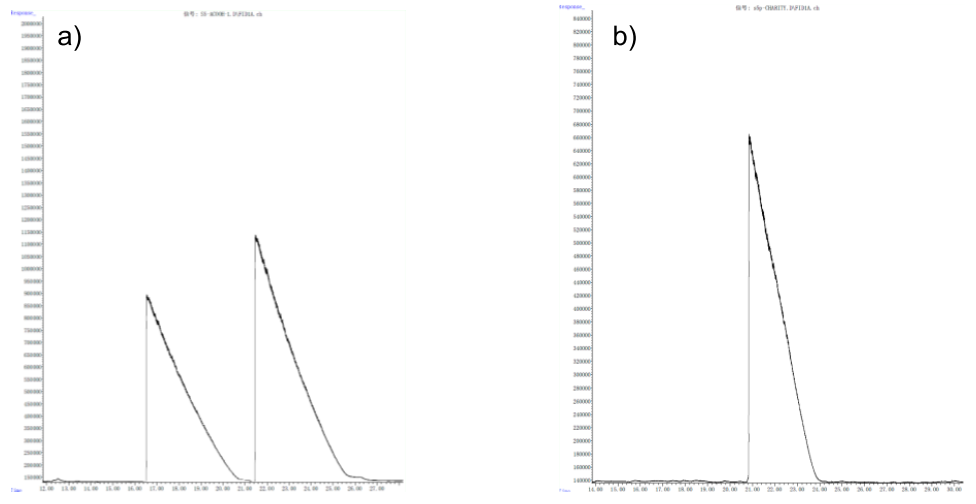
**

**Figure S12.** a) Baseline-separated enantiomers of racemic 3-methyl-δ-valerolactone (E1 *t*R = 17.3 min, E2 *t*R = 22.8 min); b) The achiral-phase GC analysis of the synthesized product. Detection conditions was described in method 1.

**Chiral value of 8b detection by HPLC**

**
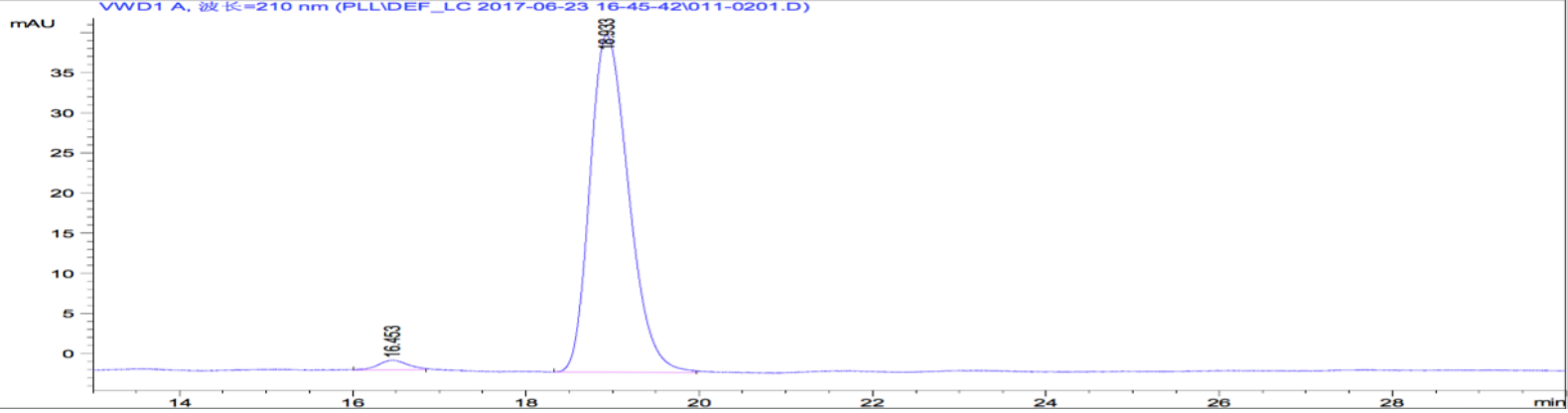
**

**Figure S13.** The achiral-phase HPLC analysis of the synthesized (R *t*R = 16.4 min, S *t*R = 18.9 min). Detection conditions was described in method 4.

**Chiral value of 9b detection by HPLC**

**
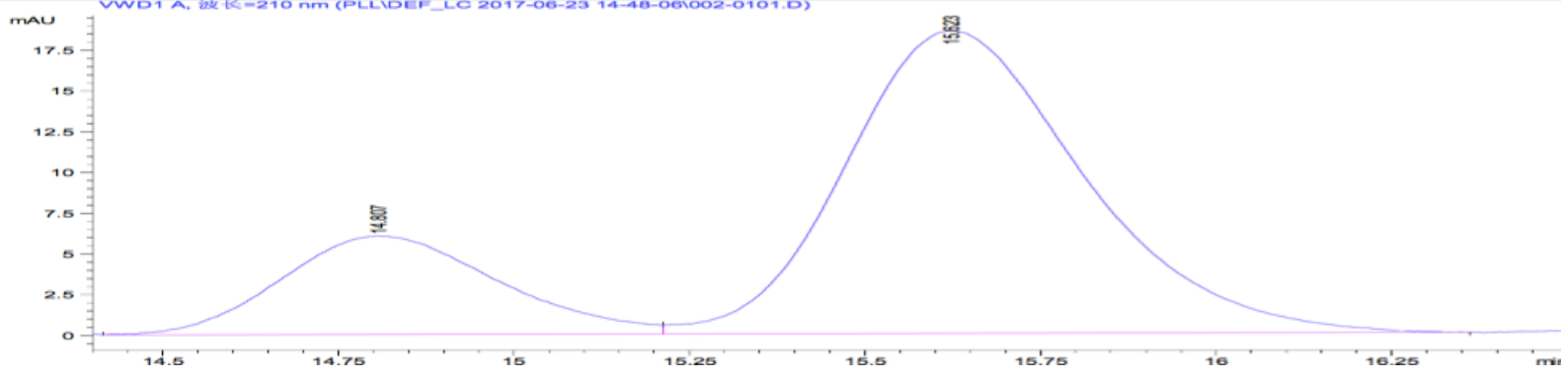
**

**Figure S14.** The achiral-phase HPLC analysis of the synthesized (R *t*R = 14.8 min, S *t*R = 15.6 min). Detection conditions was described in method 4.

**HLADH-SBFC-catalyzed oxidation of 1,4-BD in** a two-liquid phase system

**
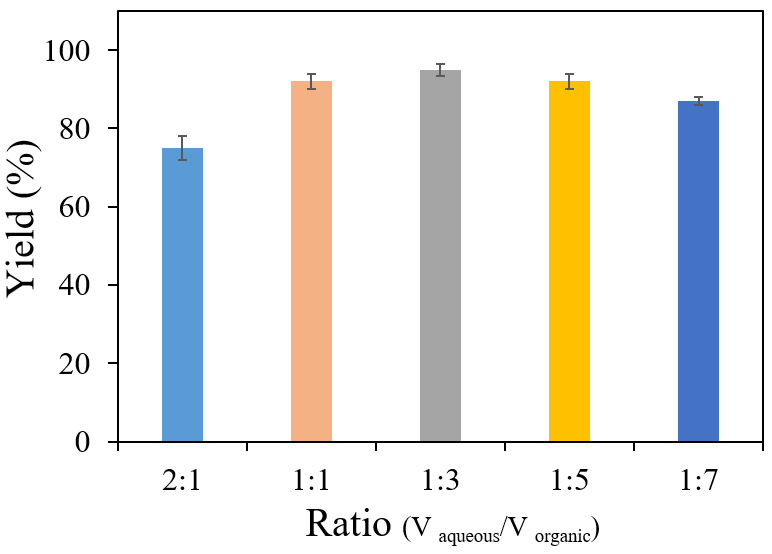
**

**Figure 15** Effect of ratio (V aqueous/V organic) on reaction

**Figure 15.** Reaction conditions: 1,4-BD (100 mM), NAD^+^ (0.1 mM), **SBFC** (0.05 mM), HLADH (0.3 g∙L^-1^), and catalase (20 U/mL) in 1 mL (50 mM) of aqueous Tris-HCl buffer (pH 8.0) at 30 °C. a) Detection after 24 h.

**Spectrum**
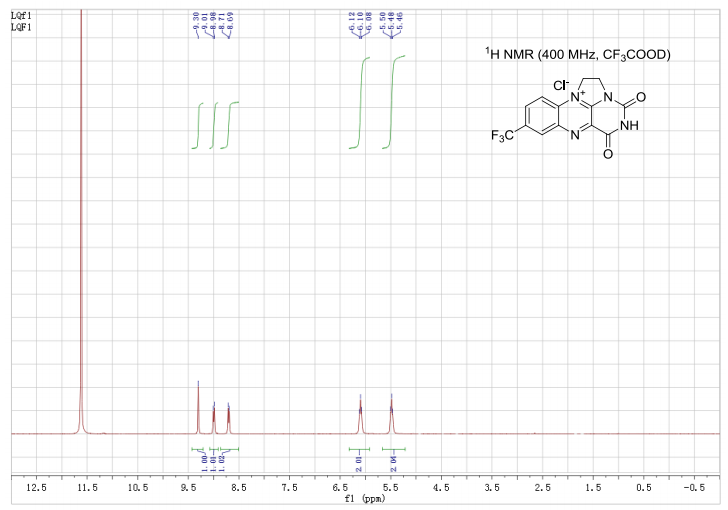


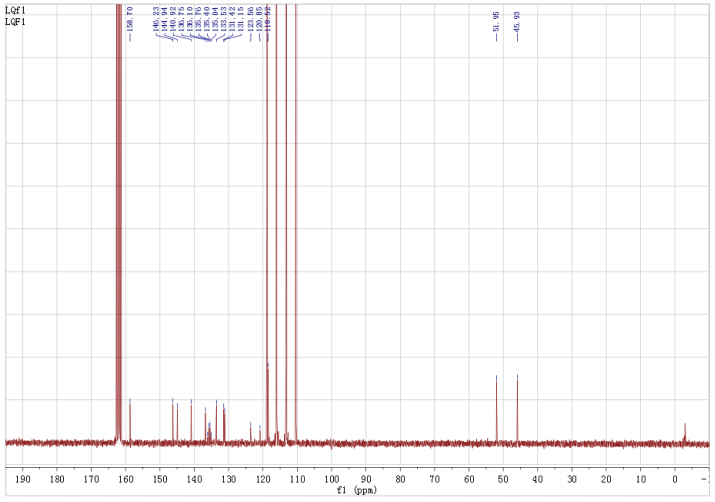


**Figure S16.** ^1^H, ^13^C of **SBFC**.

**
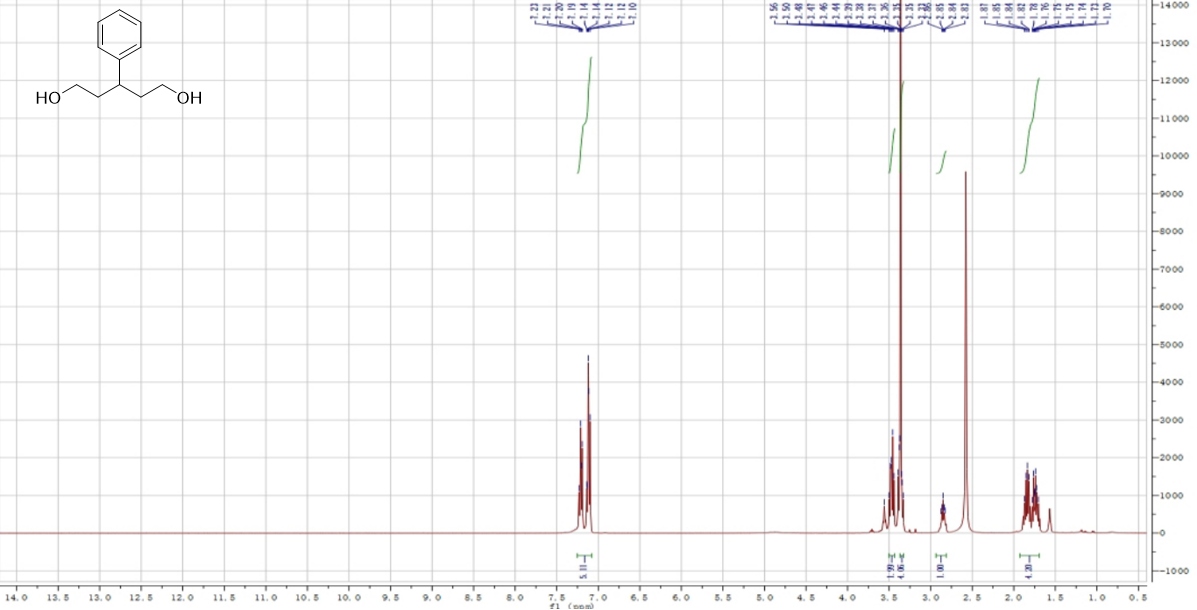
**

**
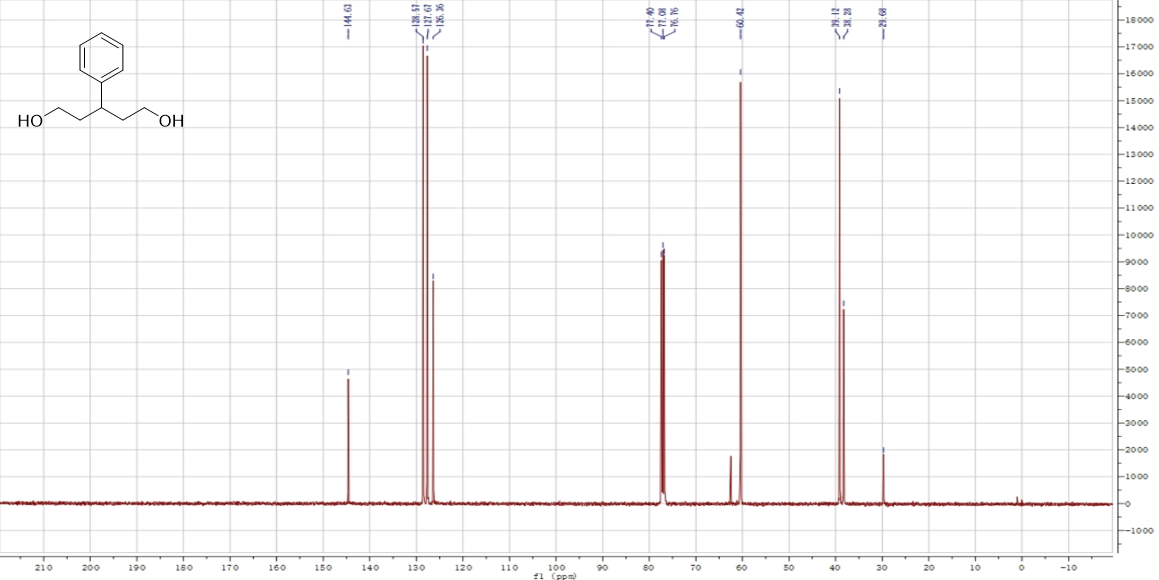
**

**Figure S17.** ^1^H, ^13^C of 8a.

**
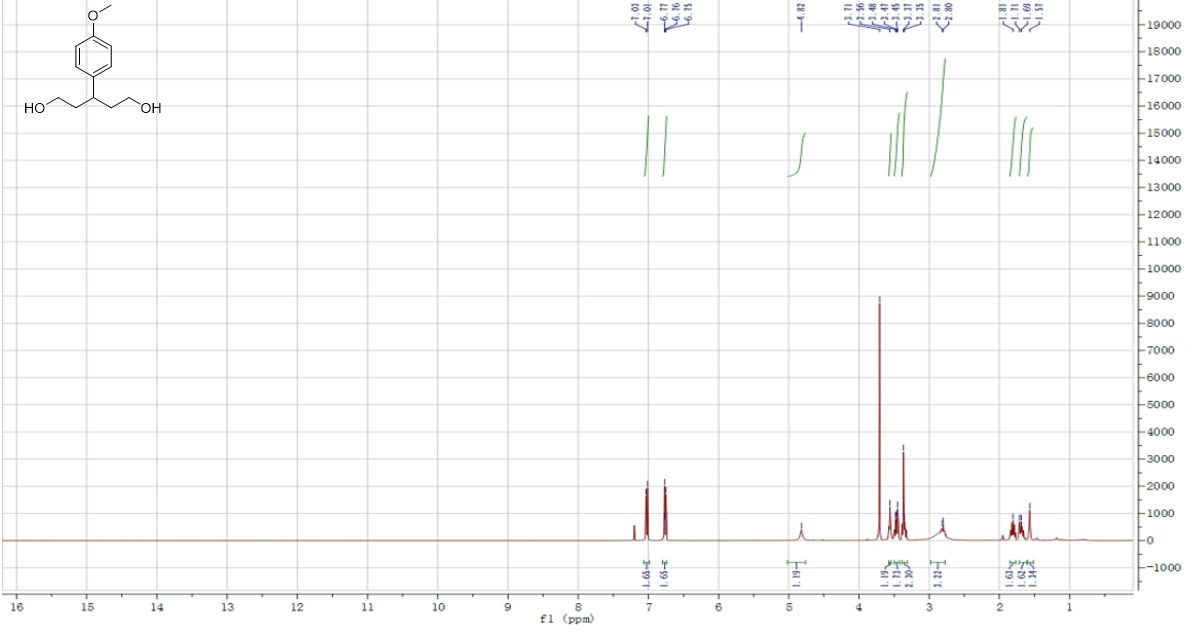
**

**
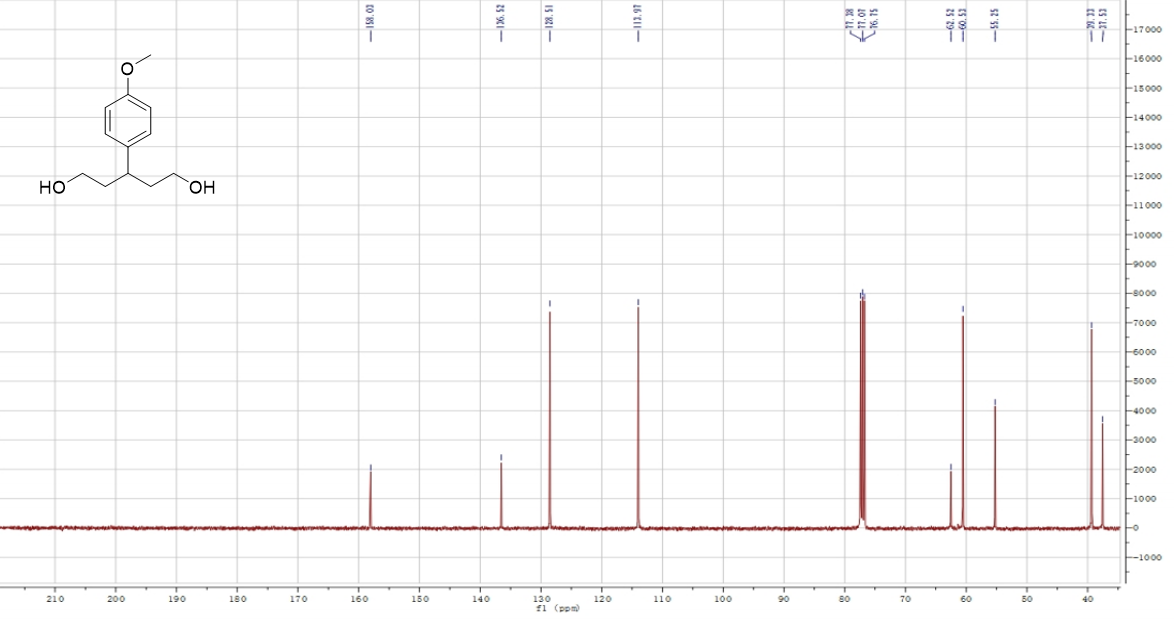
**

**Figure S18.** ^1^H, ^13^C of 9a

**Additional Tables**

**Table S1. Extraction efficient of organic solvents for** **butyrolactone and SBFC.**

| Entry | Organic  Solvent | Extraction Efficient^[a]^  Butyrolactone SBFC | |
| --- | --- | --- | --- |
| 1 | EtOAc | 0.65 | 0.2 |
| 2 | Toluene | 0.40 | 0.01 |
| 3 | Ether | 0.19 | 0.04 |
| 4 | DIPE | 0.24 | 0.01 |
| 5 | MTBE | 0.34 | 0.03 |

[a] Extraction efficient was the ratio of butyrolactone and SBFC partitioned in the extraction phase and the total butyrolactone in the biphasic system. The ratio of phases was kept to V_organic_/V_aqueous_ = 5/1.
